# Genetics of Gland-*in-situ* or Hypoplastic Congenital Hypothyroidism in Macedonia

**DOI:** 10.3389/fendo.2020.00413

**Published:** 2020-07-14

**Authors:** Nikolina Zdraveska, Mirjana Kocova, Adeline K. Nicholas, Violeta Anastasovska, Nadia Schoenmakers

**Affiliations:** ^1^Medical Faculty, University Children's Hospital, Skopje, Macedonia; ^2^University of Cambridge Metabolic Research Laboratories, Wellcome-MRC Institute of Metabolic Science, University of Cambridge, Cambridge, United Kingdom

**Keywords:** congenital hypothyroidism, dyshormonogenesis, goiter, thyroid hypoplasia, iodine, genes, screening

## Abstract

Neonatal screening in Macedonia detects congenital hypothyroidism (CH) with an incidence of 1 in 1,585, and more than 50% of cases exhibit a normally located gland-*in-situ* (GIS). Monogenic mutations causing dyshormonogenesis may underlie GIS CH; additionally, a small proportion of thyroid hypoplasia has a monogenic cause, such as *TSHR* and *PAX8* defects. The genetic architecture of Macedonian CH cases has not previously been studied. We recruited screening-detected, non-syndromic GIS CH or thyroid hypoplasia cases (*n* = 40) exhibiting a spectrum of biochemical thyroid dysfunction ranging from severe permanent to mild transient CH and including 11 familial cases. Cases were born at term, with birth weight >3,000 *g*, and thyroid morphologies included goiter (*n* = 11), thyroid hypoplasia (*n* = 6), and apparently normal-sized thyroid. A comprehensive, phenotype-driven, Sanger sequencing approach was used to identify genetic mutations underlying CH, by sequentially screening known dyshormonogenesis-associated genes and *TSHR* in GIS cases and *TSHR* and *PAX8* in cases with thyroid hypoplasia. Potentially pathogenic variants were identified in 14 cases, of which four were definitively causative; we also detected digenic variants in three cases. Seventeen variants (nine novel) were identified in *TPO* (*n* = 4), *TG* (*n* = 3), *TSHR* (*n* = 4), *DUOX2* (*n* = 4), and *PAX8* (*n* = 2). No mutations were detected in *DUOXA2, NIS, IYD*, and *SLC26A7*. The relatively low mutation frequency suggests that factors other than recognized monogenic causes (oligogenic variants, environmental factors, or novel genes) may contribute to GIS CH in this region. Future non–hypothesis-driven, next-generation sequencing studies are required to confirm these findings.

## Introduction

Congenital hypothyroidism (CH) is traditionally divided into dysgenesis (abnormal thyroid development) and dyshormonogenesis, where thyroid hormone biosynthesis is inadequate despite a structurally normal or goitrous thyroid. Shortly after the implementation of CH neonatal screening, studies investigating the incidence of CH generally reported an incidence of approximately 1:3,000, of which the majority was due to thyroid dysgenesis, with only 20% occurring due to dyshormonogenesis. More recent studies have reported a doubling in the incidence of CH, largely due to increased diagnosis of cases with a normally located gland-*in-situ* (GIS CH). Thyroid dysgenesis usually occurs because of an ectopic thyroid or athyreosis, and thyroid hypoplasia remains the least common thyroid developmental abnormality (1–3).

The etiology of GIS CH is generally unclear; however, some cases may harbor mutations in genes involved in thyroid hormone biosynthesis or development. Dyshormonogenic CH may occur because of biallelic mutations in anion transporters mediating thyroidal iodide uptake or efflux (SLC5A5/NIS or SLC26A4/pendrin, respectively); SLC26A7; TPO (the thyroid peroxidase enzyme), which catalyzes organification of iodide and the formation of thyroid hormones; and thyroglobulin (TG), upon which thyroid hormone biosynthesis and storage occur. Additional causes include monoallelic and biallelic mutations in the NADPH-oxidase DUOX2, which generates thyroidal hydrogen peroxide; its accessory protein DUOXA2; and IYD, which recycles unused iodide (1, 4, 5). Mutations in TSHR, the G-protein–coupled receptor for thyroid-stimulating hormone (TSH) cause a spectrum of phenotypes ranging from severe thyroid hypoplasia to a normal-sized GIS, with the severity correlating with the number of mutated *TSHR* alleles and the degree of receptor functional impairment (6). Thyroid dysgenesis infrequently has a monogenic basis. Isolated thyroid hypoplasia may occur because of *PAX8* or *TSHR* mutations, and mutations in the transcription factors *NKX2-1* and *FOXE1* cause CH in association with more extensive developmental syndromes (1).

The Republic of North Macedonia is a multiethnic country comprising a majority of Macedonians of Slavic origin at 64.2, 25.2% ethnic Albanians, 2.7% Roma, 3.9% Turks, and 2.2% of other ethnicities (7). Neonatal screening for CH has been mandatory in Macedonia since 2007, following a 5-year pilot study from 2002 to 2006, which included only the largest birth centers in the country. A single screening center within the University Children's Hospital in Skopje conducts the national screening program, accepting approximately 24,000 neonatal samples annually. A total of 295,909 newborns were screened between 2002 and 2017 with a mean coverage of 97.03%. Whole-blood TSH was measured from filter paper blood spots (Whatman 903), sampled 48 to 72 h after birth. The TSH cutoff level was 15 mU/L in the period 2002–2010, and 10 mU/L thereafter, which resulted in an increased prevalence of primary CH in Macedonia, from 1/2,489 live births before 2010 to 1/1,585 from 2011 onward (an increment of 36.3%) (8). A total of 153 patients were diagnosed with primary CH over this 15-year period (8–10). At reevaluation, 48.4% had permanent hypothyroidism, 30% transient CH, and 21.6% isolated hyperthyrotropinemia. Almost all cases with isolated hyperthyrotropinemia or transient CH had a normally located GIS except for two cases with isolated hyperthyrotropinemia and gland hypoplasia. Fourteen percent of permanent CH cases had likely dyshormonogenesis, and 86% had thyroid dysgenesis, of which athyreosis was the most prevalent form (53%), followed by thyroid ectopia (30%), and thyroid hypoplasia (17% of thyroid dysgenesis cases).

No studies have yet been performed to investigate the genetic basis of CH in Macedonia, despite the high percentage of GIS CH cases with potential genetically mediated dyshormonogenesis. Here, we investigated cases with non-syndromic GIS CH and normal-sized, goitrous or hypoplastic thyroid glands, either diagnosed over the aforementioned period or recruited prospectively. We used a comprehensive, sequential, targeted Sanger sequencing approach, aiming to delineate the role of mutations in known dyshormonogenesis-associated genes, *TSHR* and *PAX8*, in CH cases in this multiethnic Macedonian population.

## Materials and Methods

### Cohort Selection

The study was approved by Cambridge South REC (MREC 98/5/24) and the ethical committee at Medical Faculty, University Ss Cyril and Methodius in Skopje, and includes additional measurements undertaken as part of routine clinical follow-up with written, informed consent from patients and/or next of kin.

Congenital hypothyroidism cases born at term (*n* = 40) were selected for investigation if specific genetic causes of CH were suspected because of (1) scintigraphic features of dyshormonogenesis, (2) goiter, (3) familial occurrence of CH, (4) thyroid hypoplasia, and/or (5) cases with unexplained transient CH or isolated hyperthyrotropinemia. We did not recruit cases with athyreosis or ectopy because these individuals were deemed less likely to have an identifiable genetic cause, and labor and cost considerations precluded expansion of case numbers or inclusion of additional genes in our Sanger sequencing–based study. Screening-detected primary CH required TSH >10 mU/L and low or normal total T4 (T4) or free T4 (FT4) on confirmatory serum measurements. All children with GIS CH aged ≥3 years underwent a trial of treatment withdrawal for 4 weeks and were classified as follows: permanent CH (TSH >10 mU/L, subnormal total or FT4), transient CH (normal thyroid tests after the trial off therapy and at least 6 months' follow-up period), and isolated hyperthyrotropinemia (TSH values between 5 and 10 mU/L with normal total or FT4). We recruited cases seen in our clinics over a 1-year period and studied 34 cases with a normally located or hypoplastic GIS out of a total of 100 eligible cases from the original cohort, as well as six additional patients recruited prospectively, who did not undergo a trial off levothyroxine. Many of the original 153 cases were no longer under pediatric care or had been lost to follow-up because of emigration, and a small minority did not consent to participate.

### Prioritization of Genes for Sanger Sequencing

**Figure 1:**
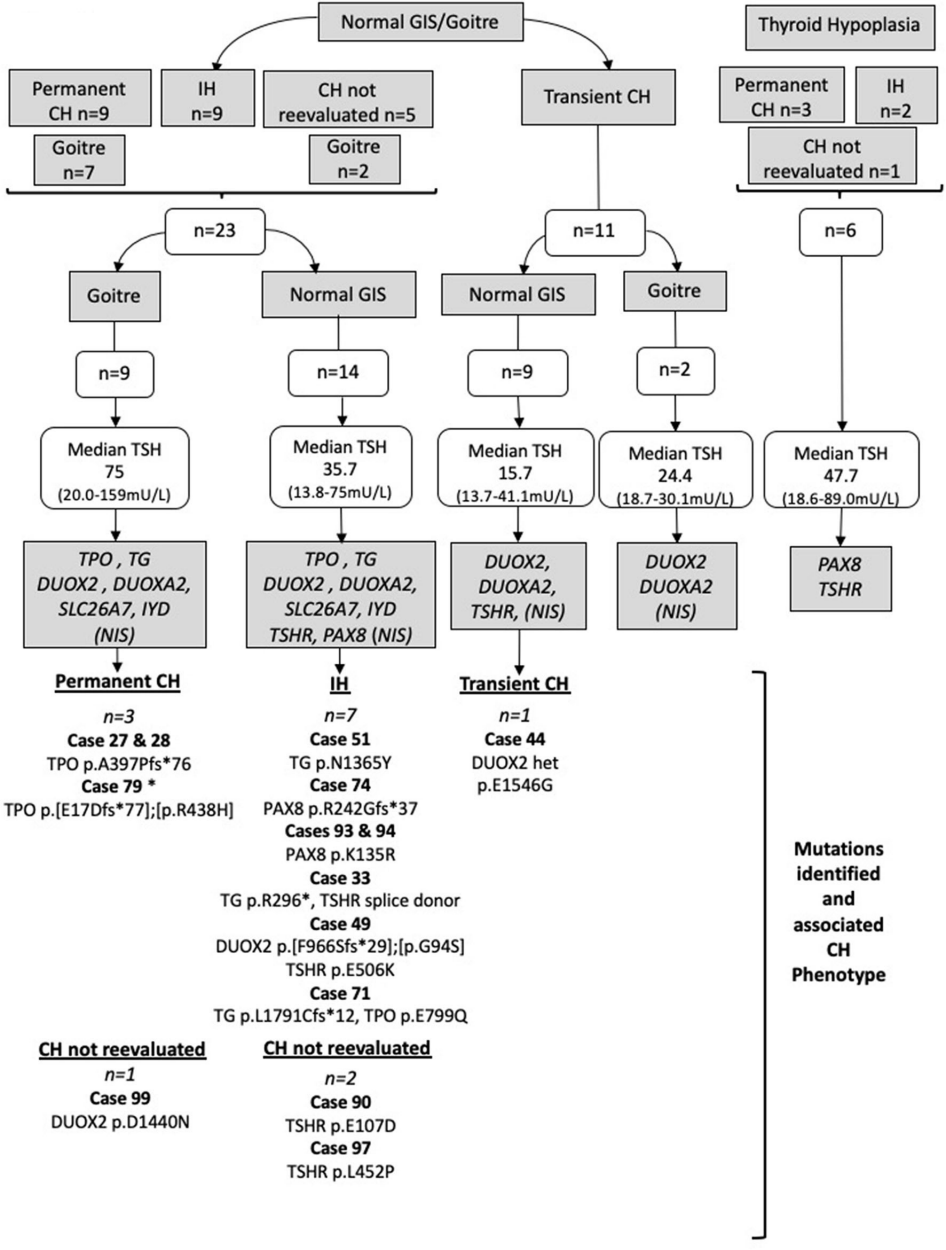
Strategy by which Sanger sequencing was used to screen individual genes most likely to be implicated in the pathogenesis of CH in particular phenotypic subgroups. Genes in gray boxes denote the genes sequenced in each subgroup. *SLC5A5* (NIS) was sequenced only in cases lacking pertechnetate scan data; otherwise, normal isotope uptake was assumed to indicate preserved NIS function. TSH values represent the median and (minimum-maximum) range; cases with TSH >75 mU/L were said to have TSH 75 mU/L for the purposes of these calculations. Cases harboring possible pathogenic genetic variants are listed for each CH category. All mutations were heterozygous or compound heterozygous. IH, isolated hyperthyrotropinemia; GIS, normal-sized, normally located gland-*in-situ*. Case 79 was presumed to have permanent CH based on levothyroxine dose requirements and the presence of goiter. Cases in whom CH was not reevaluated refer to those in whom a trial of levothyroxine withdrawal was not undertaken.

Genes were screened sequentially by Sanger sequencing following the strategy outlined in Figure 1 and summarized below, using phenotypic data (CH outcome and thyroid morphology) to predict the genes most likely to harbor mutations in the individual cases. Once a robust, likely causative variant was found, no further genes were sequenced. In cases where there was more than one affected family member, only one affected individual was sequenced, and the affected relative was genotyped if a likely causative variant was identified. Familial cases included eight siblings (four pairs) and two affected cousins (one pair) with likely autosomal recessive inheritance and an additional case with an affected father in whom we hypothesized that inheritance would be autosomal dominant.

**Normal-sized GIS/goiter (*n* =** **23) with either permanent CH (*n* =** **9), idiopathic hyperthyrotropinemia (*n*** **=** **9), or CH that was not reevaluated (*n* =** **5)**: *TG, TPO, DUOX2, DUOXA2, IYD*, and *SLC26A7* were screened, in addition to *SLC5A5* (NIS) in cases lacking pertechnetate scan data. Otherwise normal isotope uptake was assumed to indicate preserved NIS function. *TSHR* and *PAX8* were also screened in cases with a normal-sized GIS (*n* = 14) but not in goitrous cases (*n* = 9).

#### Transient CH (*n =* 11)

**Cases with normal-sized GIS (*n* =** **9)**: *TSHR, DUOX2*, and *DUOXA2* were screened. **Cases with goiter (*n*** **=** **2)**: *DUOX2* and *DUOXA2* were screened. In cases for whom pertechnetate scan data were lacking, *SLC5A5* (*NIS*) was also screened, unless uptake was normal in a sibling (one sibling pair) or there was insufficient DNA (*n* = 1 case).

**Cases with thyroid hypoplasia (*n* =** **6) with either permanent CH (*n* =** **3), idiopathic hyperthyrotropinemia (*n*** **=** **2), or CH that was not reevaluated (*n* =** **1):** *TSHR* and *PAX8* were screened irrespective of TSH level and CH outcome, because mutations in these genes are well-recognized causes of isolated thyroid hypoplasia, and the six cases all had CH without extrathyroidal manifestations. *SLC26A4* was not sequenced, because none of our cohort exhibited sensorineural deafness suggesting Pendred syndrome.

### Sanger Sequencing

Genomic DNA was extracted from peripheral blood leukocytes using standard techniques. All coding exons and exon/intron boundaries for the genes of interest were amplified by polymerase chain reaction using specific primers (available on request). Wherever possible, family members were also genotyped for mutations identified in the CH cases. Polymerase chain reaction products were sequenced using the BigDye Terminator v3.1 Cycle Sequencing Kit (Applied Biosystems, Foster City, CA, USA) and 3730 DNA Analyzer (Applied Biosystems). Variants were described using the systematic nomenclature approved by the Human Genome Variation Society (www.hgvs.org/mutnomen). Nucleotide numbering starts from A (+1) of the translation initiation codon (ATG) of the NCBI reference sequences below and includes the signal peptide where present: NM_001206744, NP_001193673 (TPO), NM_003235, NP_003226 (TG), NM_014080, NP_054799 (DUOX2), NM_207581, NP_997464 (DUOXA2), NM_000453, NP_000444 (SLC5A5), NM_203395, NP_981932 (IYD) and NM_000369, NP_000360 (TSHR), NM_003466.4, NP_003457.1 (PAX8), NM_052832.4, NP_439897.1 (SLC26A7).

### Variant Analysis

Variants with global minor allele frequency (MAF) <0.01 and maximal minor allele frequency <0.02 in the ExAC database (http://exac.broadinstitute.org) were assessed using the *in silico* pathogenicity prediction tools PolyPhen-2, SIFT, and MutationTaster. SIFT and PolyPhen-2 scores were calculated using Ensembl Variant Effect Predictor where possible or PROVEAN (for SIFT) (11–14). This relatively high maximal MAF cutoff was selected because it is recognized that certain mutations, especially those in *DUOX2*, may occur with relatively high population frequencies (MAF ≥0.01) in individuals of particular ethnicities, for example, DUOX2 p.F966Sfs^*^29 (15). Novel mutations were defined as those not present in HGMD Professional (HGMD® (http://www.hgmd.cf.ac.uk) and not detected during an NCBI PubMed search for genetic studies in CH (16).

We used three *in silico* pathogenicity predictors to assess whether variants with maximal MAF <0.02 were likely to be pathogenic and classified them according to the American College of Medical Genetics Guidelines. Variants were only considered to be possible contributors to CH if they were predicted damaging by one or more predictors, that is, predicted to be at least “possibly damaging” or “probably damaging” with PolyPhen-2 or “damaging” with SIFT or “disease causing” with MutationTaster. Given that no functional studies were undertaken in our study, and recognizing the limitations of *in silico* analysis, we also performed a literature search for known mutations in order to inform further classification of the variants.

We classified causative variants as those for which the inheritance pattern in the patient was consistent with causality and for which there was robust evidence from the literature supporting pathogenicity or where the mutations resulted in a truncated protein. Variants of uncertain significance were deemed to be possibly contributing to CH if we could not be confident that they were benign on the basis of these analyses. These data are presented in Table 1 but discussed in greater detail in the *Results* and *Discussion*.

**Table 1:** Genetic variants in mutation-positive cases.

**Case ID no**.	**TSH (mU/L)**	**Anti-TPO negative: <10 IU/mL**	**CH outcome, thyroid size**	**Gene**	**cDNA variant**	**Protein variant**	**Zyg**	**Max MAF (ExAC)**	**PP2**	**SIFT**	**MT**	**ACMG**
**Cases with likely causative variants**												
44	14.1	ND	*Tr, GIS*	*DUOX2*	c.4637A>G	p.E1546G	Het	0.0014	PD	D	DC	LP
49	50	Negative	*IH, GIS*	^*^TSHR	c.1516G>A	p.E506K (M)	Het	1.5 × 10^−5^	PD	D	DC	VUS
				*DUOX2*	^*^c.280G>A	p.G94S (M)	Compoundhet	—	PD	D	DC	VUS
					c.2895_2898 delGTTC	p.F966Sfs^*^29 (F)		0.012	PD	D	DC	P
74	16.5	Negative	*IH, GIS*	^*^PAX8	c.724delC	p.R242Gfs^*^37 (M)	Het	—	NA	NA	DC	P
79	159	ND	*Presumed Per, Goiter*	*TPO*	c.31_50dup20	p.E17Dfs^*^77 (F)	Compound het	3 × 10^−5^	NA	NA	DC	P
					c.1313G>A	p.R438H (M)		—	PD	D	DC	LP
**Cases with possible contributory variants**												
27	>75	**Negative**	***Per, Goiter***	***TPO***	**c.1184_1187** **dupGCCG**	p.A397Pfs^*^76 (F)	**Het**	—	**NA**	**NA**	**DC**	**P**
28	>75	**Negative**	***Per, Goiter***	***TPO***	**c.1184_1187** **dupGCCG**	p.A397Pfs^*^76 (F)	**Het**	—	**NA**	**NA**	**DC**	**P**
33	13.8	Negative	*IH, GIS*	^*^TSHR	c.692 + 1_692 + 4delGTGA (splice donor)	NA (M)	Het	—	NA	NA	DC	VUS
				*TG*	c.886C>T	p.R296^*^ (M)	Het	0.0008	NA	NA	DC	P
51	57.7	Negative	*IH, GIS*	^*^TG	c.4093A>T	p.N1365Y	Het	0.0002	PD	D	DC	VUS
71	14.1	ND	*IH, GIS*	^*^TG	c.5372delT	p.L1791Cfs^*^12 (F)	Het	—	NA	NA	DC	P
				^*^TPO	c.2395G>C	p.E799Q (M)	Het	—	PD	D	DC	VUS
90	13.8	ND	*NR, GIS*	^*^TSHR	c.321A>C	p.E107D (M)	Het	—	PD	T	DC	VUS
**93**	**30.3**	**ND**	***IH, GIS***	***PAX8***	**c.404A>G**	**p.K135R**	**Het**	**0.008**	**PD**	**T**	**DC**	**VUS**
**94**	**22**	**ND**	***IH, GIS***	***PAX8***	**c.404A>G**	**p.K135R**	**Het**	**0.008**	**PD**	**T**	**DC**	**VUS**
97	66.3	Negative	*NR, GIS*	^*^TSHR	c.1355T>C	p.L452P (F)	Het	1.5 × 10^−5^	PD	D	DC	VUS
99	21	Negative	*NR, Goiter*	^*^DUOX2	c.4318G>A	p.D1440N (M)	Het	3.0 × 10^−5^	PD	D	DC	VUS

*Zyg, zygosity; max. MAF, maximal minor allele frequency in the ExAC database (European non-Finnish population in all cases); TSH, venous TSH at diagnosis; (M) maternally inherited; (F) paternally inherited; B, benign; PD, probably damaging; DC, disease causing; T, tolerated; P, pathogenic; LP, likely pathogenic; VUS, variant of uncertain significance; bold, siblings; ACMG, American College of Medical Genetics classification; MT, MutationTaster; PP-2, PolyPhen-2*.

* *novel variant; Tr, Transient CH; IH, Isolated hyperthyrotropinemia; Per, Permanent CH; NR, CH not reevaluated for permanency; ND, not done; GIS, normal-sized; normally located gland-in-situ. Gray: variants in oligogenic cases that are possibly contributing to CH, in addition to variants which are more likely to be causative*.

### Urinary Iodine Measurement

Twenty-four-hour urinary iodide excretion was measured at the National Laboratory for Determining Iodine in Urine using method A [World Health Organization (WHO)/United Nations International Children's Fund (UNICEF)/International Council for the Control of Iodine Deficiency Disorders (ICCIDD)] with ammonium persulfate solution and spectrophotometric detection of the Sandell–Kolthoff reaction (Spectrophotometer UNICO UV-2102, USA). The results of urine iodide excretion were interpreted in accordance with WHO recommendations (school-aged children: adequate iodide: 100–199 μg/L, iodide excess >300 μg/L; children aged <2 years: adequate iodide ≥100 μg/L, insufficient iodide ≤ 100 μg/L).

### Thyroid Imaging

Thyroid pertechnetate scans were performed at diagnosis using a double-head Mediso gamma camera after an intravenous injection of 99 m-technetium pertechnetate (2 MBq/kg), obtaining standard anterior and lateral images and recording the size and location of areas of 99 m TcO_4_ uptake. Thyroid ultrasound (SonoScape SSI-5000 Color Doppler Ultrasound System; SonoScape Medical Corp., Shenzhen, China) was performed by a single experienced physician at diagnosis, comparing the volume of the thyroid lobes with reference values from the literature.

### Statistical Analysis

Statistical analyses were performed using Prism (GraphPad Software Inc., USA), version 5.0 d.

### Biochemical Assays

Serum TSH and T4/FT4 and anti–thyroid peroxidase autoantibodies (anti-TPO) were measured locally using the IMMULITE 2000 chemiluminescent enzyme immunoassay system (Siemens Healthcare Diagnostics Inc., USA). Thyroid hormone levels falling within the 3rd to the 97th percentile standardized for age were considered normal. The reference ranges for anti-TPO antibodies were 10–35 IU/mL.

## Results

### Description of Cohort

Figure 1 summarizes the genetic evaluation undertaken, in addition to median TSH results and imaging data for the 40 cases. Overall, median serum TSH at diagnosis was 30.3 mU/L (range, 13.7–159 mU/L); the thyroid gland was ultrasonographically normal sized in 57.5% of cases, goitrous in 27.5%, and hypoplastic in 15% of cases. The outcome of the CH was determined in 85% of cases, and equal proportions demonstrated permanent and transient hypothyroidism and isolated hyperthyrotropinemia. Eleven cases (27.5%) had familial CH with at least one affected relative. In three cases, total T4 was elevated at diagnosis, but FT4 was normal with elevated TSH, supporting a diagnosis of CH with a possible binding protein abnormality.

### Mutation Positive Cases

Fourteen patients harbored rare genetic variants (Table 1), which had either been associated with CH in previous studies or which were novel but predicted to be pathogenic by at least one *in silico* predictive tool (PolyPhen-2, SIFT, or MutationTaster). Cases were subclassified according to the variants found, with only four cases felt to harbor definitive, causative variants. Ten patients harbored variants that were possibly contributing to CH, but either there was insufficient evidence to label the variants as causative, or the zygosity of the variants did not support sole causality (e.g., heterozygous *TPO* or *TG* mutations). Two of these cases harbored digenic variants. Mutation-positive cases harbored variants in *TPO, TG, TSHR, DUOX2*, and *PAX8*. We did not detect any mutations in *DUOXA2, NIS, IYD*, or *SLC26A7*.

### Monogenic Variants

Previously reported *TPO* variants were identified in three cases including two siblings who each harbored a heterozygous frameshift variant, p.A397Pfs^*^76, inherited from a euthyroid father. An unrelated case was compound heterozygous for a maternally inherited missense mutation, p.R438H, and a paternally inherited frameshift variant, p.E17Dfs^*^77. All three cases exhibited severe CH at diagnosis, with significantly elevated serum TSH values (median, >75 mU/L; range, >75–159 mU/L) and low fT4 values (median, <4 μg/dL; range, <4–1.02 μg/dL). Goiter was diagnosed on the neonatal thyroid ultrasound in cases harboring p.A397Pfs^*^76 and during fetal ultrasound examination in the case with compound heterozygous mutations.

Heterozygous *DUOX2* variants were identified in two cases. One case was heterozygous for DUOX2 p.E1546G, a previously reported variant that was associated with transient CH. Parental DNA was unavailable for genotyping. The second case harbored a novel, maternally inherited variant, DUOX2 p.D1440N, associated with goiter, mildly elevated blood spot and serum TSH (14.8 and 21 mU/L, respectively), and normal FT4 1.39 ng/dL. During the first year, levothyroxine requirements were low (dose at 1 year of age 1.6 μg/kg), suggesting that the CH may also eventually resolve.

Two cases harbored heterozygous *TSHR* variants, both of which were novel. Variants p.L452P, and p.E107D, were predicted to be pathogenic by two (p.E107D) or three (p.L452P) algorithms, and a single carrier parent was identified for each variant with either symptomatic hypothyroidism (p.L452P) or isolated hyperthyrotropinemia (p.E107D). Both *TSHR* variants were associated with normal thyroid size and mild CH; because both cases were younger than 3 years, treatment withdrawal was not possible; however, both individuals readily achieved normalization of TSH on levothyroxine treatment, unlike some reported cases (usually with biallelic mutations) in whom TSH normalization has required supraphysiological FT4 levels (17).

Heterozygous *PAX8* mutations were identified in three cases, two of whom were siblings. All had isolated hyperthyrotropinemia with a normal-sized GIS. The first case harbored a frameshift mutation (p.R242Gfs^*^37), which is likely causative, and was inherited from her heterozygous mother who was also on levothyroxine for hypothyroidism. The missense variant p.K135R in the siblings was less definitive, and parental DNA was unfortunately not available for analysis. Although previously reported in association with CH, it has not been functionally characterized and is predicted to be tolerated by SIFT (18). A novel, heterozygous *TG* variant of uncertain significance (p.N1365Y) was identified in one further case, which is unlikely to be causative in isolation because TG variants associated with CH are usually biallelic.

### Oligogenic Variants

**Figure 2:**
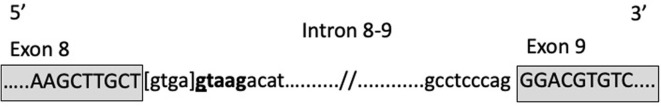
Graphic description of the TSHR donor splice site variant in case 33. [] means deleted bases; bold font indicates splice site after deletion.

Three cases with isolated hyperthyrotropinemia harbored oligogenic variants, although it is not clear whether all played a causal role in CH. The first case harbored compound heterozygous DUOX2 mutations (p.G94S, p.F966Sfs^*^29), of which p.F966Sfs^*^29 is a known pathogenic mutation and likely to be the main cause for CH in this case. Although a coexisting, heterozygous TSHR variant (p.E506K) was identified, which was strongly predicted to be pathogenic, it was inherited from a euthyroid mother. A second case harbored both a novel, heterozygous TPO mutation (p.E799Q) affecting the same amino acid as a previously reported mutation (p.E799K) and a heterozygous TG mutation, resulting in a frameshift (p.L1791Cfs^*^12). Because biallelic TG and TPO mutations are usually required for CH to develop, the cause for CH has not yet been completely elucidated in this case. The final case harbored a known pathogenic, heterozygous TG mutation (p.R296^*^) and a *TSHR* splice donor variant, c.692 + 1_692 + 4delGTGA. The latter was unlikely to be a contributor to CH, being inherited from a euthyroid mother and retaining a canonical 5′ splice site sequence, making it plausible that normal splicing will be preserved (Figure 2), and the heterozygosity of the TG mutation also makes its contribution to CH unclear.

Variant-positive and variant-negative cohorts exhibited similar thyroid size, biochemistry, and demographic features (Table 2). Twenty-four-hour urinary iodine concentrations were assessed in seven mutation-negative cases and three mutation-positive cases and did not show iodine deficiency (range, 124–329 μg/L). A single individual with 24-h urinary iodine concentration of 329 μg/L may have been subject to excessive iodine intake; however, because this child was younger than 3 years at the time of sampling, he was too young for accurate comparison with the WHO school-aged reference ranges. Anti-TPO titers were assessed in cases with a maternal history of thyroid disease, either at diagnosis or early in the follow-up. Fifty-seven percent of the mutation-positive group and 42% of the mutation-negative group were tested, all of them showing levels below the measurement level (<10 IU/mL).

**Table 2:** Summary of mutation-positive and mutation-negative cases.

	**Mutation-positive cases**	**Mutation-negative cases**
Number of cases	14	26
Ethnicity of cases	11 Macedonian, 3 Albanian	17 Macedonian, 7 Albanian, 2 Roma
% of cohort	35	65
Familial cases	4	7
Median TSH (mU/L) and range	26.2 (13.8–159)	35.7 (13.7–152)
% cases with TSH ≥ 50 mU/L	43	31
% with goiter	29	27
% with normal-sized gland	71	50
% with hypoplastic gland	0	23
Genes harboring variants	*TPO* (*n =* 4) *TSHR* (*n =* 4) *DUOX2* (*n =* 4) *TG* (*n =* 4) *PAX8* (*n =* 3)	

*There was no significant difference in median TSH values (Mann–Whitney test p = 0.63) or proportion of cases with TSH ≥50 mU/L (χ^2^ test 0.44) or gland morphology (χ^2^ test, p = 0.14) between the two groups. Cases with TSH >75 mU/L were said to have TSH 75 mU/L for the purposes of these calculations*.

## Discussion

This is the first study investigating genetic causes of CH in Macedonian patients. In this small cohort of selected CH cases, targeted genetic analysis revealed potential pathogenic variants in *TPO, DUOX2, PAX8, TSHR*, and *TG. Seven* variants either truncated the protein, affecting key functional domains, or had previously been demonstrated to result in loss of function *in vitro*. Additionally, nine missense variants were identified in *TSHR* (*n* = 3), *TPO* (*n* = 2), *DUOX2* (*n* = 2), *PAX8* (*n* = 1), *TG* (*n* = 1), as well as a donor splice site variant in *TSHR*. *In silico* analyses, genotype–phenotype correlation and analysis of clinical phenotype were used to assess likelihood of loss of function. Future studies using functional assays with protein expression in eukaryotic cells will be essential in order to permit more definitive statements regarding pathogenicity, but were outside the scope of this study.

Macedonian cases harboring *TPO* mutations all had severe, goitrous CH, as is typical in this context, and the fetal goiter observed in one case is a rare, but recognized presentation (19). Both TPO p.A397Pfs^*^76 and p.E17Dfs^*^77 have been associated with CH and iodide organification defect, and pathogenicity of both mutations has been confirmed in thyroid tissue from affected patients (20, 21). TPO p.R438H is located within the TPO heme peroxidase catalytic domain and has been associated with severe CH, although its effects on catalytic activity have not been characterized *in vitro* (22). Congenital hypothyroidism–associated TPO mutations are usually biallelic; however, two siblings harbored a TPO mutation, which was definitively pathogenic (p.A397Pfs^*^76) but heterozygous, without an identifiable mutation on the other allele. Although heterozygous TPO mutations are occasionally associated with CH, their heterozygous father was euthyroid; therefore, it is more likely that they harbor an additional, undetected intronic or regulatory region mutation on the maternal *TPO* allele. In a similar case with total iodide organification defect and heterozygous TPO mutation, monoallelic TPO expression was demonstrated in the thyroid gland (23, 24).

Monogenic *DUOX2* mutations frequently cause goitrous transient CH or TSH resistance (15, 25). DUOX2 p.E1546G exhibits impaired H_2_O_2_ synthesis *in vitro* and has been associated with permanent CH when inherited in compound heterozygosity with the non-functional DUOX2 p.M866R (15). We identified one case who was heterozygous for DUOX2 p.E1546G alone and had non-goitrous, transient CH. Absence of the highly deleterious DUOX2 p.M866R may have contributed to this milder phenotype, although *DUOX2* genotype–phenotype correlation is poor (26). An additional case had a novel heterozygous DUOX2 variant p.D1440N affecting a conserved amino acid within the NADPH-oxidase domain in which other pathogenic mutations have been identified, for example, p.G1518S (15, 27, 28). This, together with the associated mild, goitrous CH, supports pathogenicity of the mutation.

*TSHR* mutations may underlie CH in cases with normal-sized or hypoplastic thyroid because TSHR signaling influences both thyroid growth and hormonogenesis (6). We identified two heterozygous, monogenic *TSHR* variants associated with normal thyroid size and mild CH, which could be consistent with TSHR dysfunction (p.E107D, p.L452P). Both were novel; however, their inheritance from parents with isolated hyperthyrotropinemia or overt hypothyroidism increased the likelihood of pathogenicity. Conversely, the significance of TSHR p.E506K was unclear because it did not segregate with phenotype, and *TSHR* c.692 + 1_692 + 4delGTGA was unlikely to be pathogenic because the canonical 5′ splice site sequence was preserved.

*PAX8* p.R242Gfs^*^37 encodes a protein with a preserved paired domain and octopeptide with a frameshift at the distal end of the homeodomain homology region and a truncation proximal to the C-terminal region. Experimentally validated, pathogenic *PAX8* mutations usually affect the paired domain, an exception being *PAX8*c.989_992 delACCC, which exhibits preserved binding to a paired domain recognition sequence but transcriptional inactivity, due to disruption of the C-terminal activation domain (29). *PAX8* p.R242Gfs^*^37 truncates PAX8 one amino acid downstream of c.989_992 delACCC and thus will be similarly pathogenic, with the perturbed homeodomain potentially mediating additional effects. Reported *PAX8* mutations are always heterozygous and can be associated with intrafamilial phenotype variability ranging from severe CH to euthyroidism or the isolated hyperthyrotropinemia observed in case 74. *PAX8* p.K135R identified in two siblings is more equivocal. It is located in the paired domain; although the p.K135A substitution has been shown to impair wild-type transactivation function, substitution of lysine (K) for arginine (R), which is also positively charged, may be better tolerated. There is also one homozygote for PAX8 p.K135R in the ExAC database, suggesting its functional consequences are likely to be mild despite its association with CH in a previous study (8, 30).

Mutation frequency in GIS CH is strongly influenced by cohort ethnicity and selection criteria, and genetic studies have not previously been undertaken in Macedonian CH cases. The rank order of potentially causative genes in our cohort is comparable with other studies, because *TSHR* mutations commonly cause non-autoimune hyperthyrotropinemia, *TPO* mutations frequently underlie dyshormonogenesis, and *DUOX2* mutations are a common cause of either mild/transient CH or TSH resistance (6, 25, 28, 31, 32). Surprisingly, although biallelic *TG* mutations are a frequent cause of CH, only monoallelic mutations were identified in our study, which may reflect the relatively small number of cases screened. Although TG p.L1791Cfs^*^12 and p.R296^*^ are pathogenic, both were inherited in double heterozygosity with the novel TPO variant p.E799Q and the likely benign *TSHR* variant c.692 + 1_692 + 4delGTGA, respectively; therefore, the relative contributions of the two mutations are not clear, and it is also possible that an undetected, non-exonic TG mutation on the other allele is contributing to CH. The significance of TG p.N1365Y in case 51 is equivocal.

No *SLC26A7, DUOXA2, NIS*, or *IYD* mutations were identified. Mutations in the latter three genes are generally rare; however, because *SLC26A7* has not yet been sequenced in large CH studies, the frequency of CH-associated mutations is unknown. Larger studies, with an unselected sequencing approach, are needed to compare genotype–phenotype correlations in multiethnic Macedonian CH cases and with CH populations of different ethnicities. There was no significant difference in thyroid biochemistry or morphology between the mutation-positive and mutation-negative cases.

Sixty-five percent of our cohort did not harbor a likely causative mutation, and 79% of our cohort had a normal T4 at diagnosis; therefore, CH was generally mild–moderate. The incidence of mild GIS CH is increasing, and a previous next-generation sequencing (NGS) study in such cases found that biochemically mild CH was less likely to have an identifiable genetic basis, consistent with our low rate of definitively causal mutations (33). However, a major limitation to our study is the fact that we used a phenotype-driven approach, sequentially screening individual candidate genes, because (as in many diagnostic settings) an NGS-based panel was not readily available to us. This approach may have been inadequate for elucidating the basis of all genetically mediated CH cases.

Next-generation sequencing is now the recommended approach for evaluating CH, using either a candidate gene panel or whole-exome (WES) or genome sequencing, and permits a non–hypothesis-driven approach, additionally detecting mutations in CH-associated genes associated with an unexpected phenotype (34, 35). This is important both for GIS CH and thyroid dysgenesis because variants in genes involved in thyroid hormone biosynthesis (e.g., *Pendrin, TPO*, and *DUOX2*) have recently been implicated in thyroid dysgenesis, and mutations in morphogenesis-associated genes (e.g., *FOXE1*) have been implicated in GIS CH (18, 34–38). A further advantage of NGS is the ability to detect oligogenic variants, because cumulative effects of rare variants in CH-associated genes that confer minor functional impairment individually have been implicated in development of both GIS CH and thyroid dysgenesis. Oligogenicity may also modulate CH phenotype explaining the variable penetrance and expressivity of genetic defects observed in familial CH (18, 28, 34, 39, 40). Finally, WES and whole-genome sequencing have enabled novel genetic causes of CH to be defined, such as *SLC26A7* mutations causing dyshormonogenesis, and *TUBB1* and CDCA8 mutations in dysgenesis (4, 41, 42).

Our phenotype-driven approach may have missed CH-associated variants in cases with atypical phenotypes, especially in cases with transient CH or thyroid hypoplasia where the sequencing was least comprehensive. Additionally, we did not sequence *NKX2-1, FOXE1*, and *GLIS3*, which also contribute to isolated GIS CH, although affected patients often have additional extrathyroidal features (18). Although three digenic cases were found in our cohort, our Sanger sequencing approach may also have underestimated the contribution of oligogenicity to CH in our cases and will only have detected known genetic causes. Additional limitations include the fact that neonatal ultrasonography may not always have defined thyroid size accurately because of technical challenges in scanning babies, potentially resulting in inappropriate selection of genes for screening. Moreover, because <40% of the total number of Macedonian isolated hyperthyrotropinemia, transient CH, and thyroid hypoplasia cases were screened, our findings may not be representative of the entire Macedonian CH population.

Although the true genetic mutation frequency may have been higher in our cohort had we used an NGS approach, the low frequency of mutation-positive cases nevertheless suggests that novel genetic or environmental factors alone, or in concert with recognized genetic causes, may play a role in the pathogenesis of GIS CH. Five cases with goitrous CH (three familial) were not found to have a causative mutation despite comprehensive screening of all genes involved in thyroid hormone biosynthesis. Because mutations in morphogenesis-associated genes have not yet been found to cause goitrous CH to the best of our knowledge, this observation supports the existence of alternative causes for dyshormonogenic goiter. Similar mutation-negative familial or consanguineous cases have been reported in other studies, justifying a role for future exome or genome sequencing studies to investigate the occurrence of mutations in novel genes in this subset.

The etiology of transient CH in particular is also poorly ascertained in the literature with mutations in *DUOX2* and *DUOXA2* representing the best characterized genetic causes; however, only one transient CH case in our study harbored a *DUOX2* mutation, and no *DUOXA2* mutations were detected (28). Environmental contributors to transient CH include suboptimal iodine status; however, urinary iodine concentration was unfortunately ascertained in only 10 cases asynchronously with CH diagnosis. The rationale for this is that Macedonia is one of the 11 countries in the world that has continuously sustained iodine sufficiency according to the Iodine Global Network Reports (2018) (43). Consistent with this, the 10 evaluated cases were iodine-replete at assessment; however, we cannot definitively exclude an etiological role for neonatal iodide deficiency or excess, especially in cases with transient CH or isolated hyperthyrotropinemia. Additionally, the roles of thyroid disruptors such as perchlorate, thiocyanate, and nitrates in CH have not yet been evaluated.

In summary, in this first genetic evaluation of CH cases with GIS or thyroid hypoplasia in Macedonia, direct sequencing identified definitively causative variants in four cases and potentially pathogenic variants in a further 10, involving *TPO, TG, TSHR, DUOX2*, and *PAX8*, all genes that are frequently implicated in CH worldwide. Despite methodological limitations, the relatively low mutation frequency suggests that factors other than recognized monogenic causes (oligogenic variants, environmental factors, or novel genes) may contribute to GIS CH in this region, mandating future non–hypothesis-driven, NGS studies to confirm these findings.

## Data Availability Statement

The datasets for this article are not publically available due to ethical constraints. Requests by qualified researchers to access anonymized data should be directed to Dr. Nadia Schoenmakers, naaa2@cam.ac.uk.

## Ethics Statement

The studies involving human participants were reviewed and approved by Cambridge South REC (MREC 98/5/024) and the Ethical Committee at Medical Faculty, University Ss Cyril and Methodius in Skopje. Written informed consent to participate in this study was provided by the participants' legal guardian/next of kin.

## Author Contributions

All authors have accepted responsibility for the entire content of this submitted manuscript and approved submission. NZ has the main contribution in designing, writing and editing of the manuscript, searching literature, and following the patients. MK was involved in diagnosing, following and treating the patients, performing the ultrasound check-ups, designing, and writing the manuscript. AN carried out the molecular genetic studies, designed the sequencing strategy, participated in writing the manuscript. VA was responsible for the newborn thyroid screening, detecting the CH patients and editing of the manuscript. NS performed the molecular genetic studies, designed the sequencing strategy, helped in the interpretation of the results and participated in writing, and editing of the manuscript.

## Conflict of Interest

The authors declare that the research was conducted in the absence of any commercial or financial relationships that could be construed as a potential conflict of interest.
